# *Origanum majorana* Essential Oil—A Review of Its Chemical Profile and Pesticide Activity

**DOI:** 10.3390/life12121982

**Published:** 2022-11-26

**Authors:** Eleni Kakouri, Dimitra Daferera, Charalabos Kanakis, Panagiota-Kyriaki Revelou, Eleftheria H. Kaparakou, Sofia Dervisoglou, Dionysios Perdikis, Petros A. Tarantilis

**Affiliations:** 1 Laboratory of Chemistry, Department of Food Science & Human Nutrition, School of Food and Nutritional Sciences, Agricultural University of Athens, Iera Odos 75, 11855 Athens, Greece; 2 Laboratory of Agricultural Zoology and Entomology, Department of Crop Science, Agricultural University of Athens, 11855 Athens, Greece

**Keywords:** *Origanum majorana*, essential oil, natural pesticide, repellent, fumigant, insecticide, secondary metabolites, volatile profile, terpinen-4-ol, carvacrol

## Abstract

*Origanum majorana* is a medicinal and aromatic plant that belongs to the Lamiaceae family. It is cultivated in several parts of the world and, due to its splendid aroma and taste, is widely used for culinary purposes and in perfumes. The essential oil of the plant, to which is attributed its aroma, contains many secondary metabolites with valuable biological activity. One of them is the pesticide activity, which has attracted much interest. Given the necessity of replacing synthetic pesticides, essential oils are studied in an attempt to find naturally derived products. Thus, the aim of this review paper is to discuss the chemical profile of *O. majorana* essential oil and to present data regarding its insecticidal, repellent and fumigant activity. Data were collected from 1992 to 2022. Databases, including PubMed, Google Scholar, ScienceDirect and Scopus, were used for the research, and keywords, including *O. majorana*, sweet marjoram, essential oil, volatiles, pesticide, insecticide and repellent activity, were used. The results of this review paper indicate that *O. majorana* essential oil can be an alternative agent to manage pests. However, still, much research should be conducted to evaluate its toxicity against beneficial insects and to ensure its safety for human health.

## 1. Introduction

Aromatic plants are plants that produce and exude from their different plant organs (leaves, flowers, etc.) aromatic substances, which are used for cosmetic and culinary purposes. On the other hand, according to WHO, medicinal plants are defined as those plants (wild or cultivated) that contain a mixture of active compounds, able to prevent, relieve or cure diseases or serve as lead molecules for the discovery of new drug formulations. These compounds are synthesized through common biochemical pathways shared by primary and secondary metabolism and are commonly known as secondary metabolites. Plants provide a plethora of secondary metabolites that exert significant biological activity.

Lamiaceae is a family well studied for the presence of secondary metabolites, which includes volatile and nonvolatile compounds that are present as complex mixtures. These complex mixtures provide significant biological activity, making these plants useful in the food, cosmetic and pharmaceutical industries [1,2].

*O. majorana* L. belongs to the large family of Lamiaceae plants, which consists of 230 genera and almost 7000 species [3]. It is a perennial aromatic, annual herb. Its synonym and accepted botanical name is *Majorana hortensis*, while the plant is commonly known as sweet marjoram. The plant is native to Greece, Cyprus and Turkey; however, it has also been cultivated in Morocco, Egypt, Tunisia, Algeria and elsewhere [4,5].

*O. majorana* is among the well-studied species of the Lamiaceae family. Its rich chemical profile, either referring to the essential oil fraction or the extracts of the plant, has classified *O. majorana* as a plant with valuable pharmacological activities [5,6,7,8,9,10]. In particular, the biological activity of the essential oil derived from the aerial part of the plant has been examined in various studies. Many properties have been attributed to this fraction of secondary metabolites, including antioxidant, antimicrobial, anti-inflammatory, antiacetylcholinesterase, anticancer, antidepressant and analgesic [11,12,13,14,15,16,17]. Apart from the above-mentioned biological activities, the repellent and insecticidal activity of the essential oil of the plant is of maximum importance [18,19,20]. Nowadays, in order to ensure food availability, crops are treated with synthetic pesticides, for which is intensively discussed their negative impact on human health and the environment as well [21]. Biological replacements for synthetic pesticides currently in use could be essential oils [22]. Thus, a considerable number of studies examine the biological activity of essential oils as candidate pesticides against many insect species [23,24,25]. These naturally derived products aim to protect crops in an eco-friendly manner and at the same time not to adversely affect human health. In particular, regarding the essential oil of *O. majorana*, its insecticidal, larvicidal, repellent and fumigant activities have been evaluated [20,24,26,27,28,29,30,31,32]. Given the potential of the essential oil of the plant in insect pest control as revealed by the above-mentioned studies, this review aims (a) to gather information regarding the chemical profile of *O. majorana* essential oil, as has been described by various researchers from different countries; (b) to discuss its pesticide activity in an attempt to evaluate its possible use as a naturally derived insecticide, repellent or fumigant agent. Databases, including PubMed, Google Scholar, ScienceDirect and Scopus, were used for the research, and keywords, including *O. majorana*, sweet marjoram, essential oil, volatiles, pesticide, insecticide and repellent activity, were used.

## 2. Chemical Profile of *O. majorana* Essential Oil

Essential oils are complex mixtures, consisting of volatile, usually aromatic, colorless compounds, poorly soluble in water but highly soluble in many organic solvents such as acetone, ethanol and diethyl ether. They are products of the secretory system of the plants, obtained via different procedures, which depend on the plant part used. The most common isolation methods are hydrodistillation and steam distillation, applied when the essential oil is obtained from the aerial parts of the plant.

Volatiles are accumulated at the glandular trichomes of the reproductive and vegetative organs of the plants that belong to the Lamiaceae family. In particular, they are more abundant in reproductive organs and young leaves [33]. Thus, in general, the most popular parts of the plants used are stems, flowers and leaves, from which essential oil is extracted mainly by steam distillation.

Typical constituents of the essential oils are terpenoids and more precisely monoterpenes, which are flavor compounds and sesquiterpenes, oxygenated or not. Other constituents include derivates of monoterpenes, which means compounds bearing different functional groups such as esters, acetates and alcohols [34]. Monoterpenes and sesquiterpenes are indicated by the molecular formula (C_5_H_8_)n, in which *n* = 2 in the monoterpenes case since they consist of two isoprene units. On the other hand, sesquiterpenes consist of three isoprene units; thus, *n* equals 3.

For *O. majorana*, characteristic volatile compounds presented in great quantities are monoterpenes hydrocarbons and oxygenated monoterpenes. Other constituents, less in quantity, are sesquiterpenes, oxygenated or not (Table 1). As discussed below, the oxygenated monoterpenes prevail in most cases in the *O. majorana* essential oil derived from different geographical regions, with terpinen-4-ol being the most abundant compound [35,36,37,38,39,40,41,42,43,44,45,46,47,48].

In most studies, compounds detected in abundance were terpinen-4-ol, *cis*-sabinene hydrate and *γ*-terpinene, while in some cases, the essential oil is rich in carvacrol and thymol, with the concentration of terpinen-4-ol being half of carvacrol or even absent [20,48,49,50,51,52,53,54,55]. Thus, researchers have classified *O. majorana* into two main chemotypes, based on qualitative criteria. The first one is the terpinen-4-ol/cis-sabinene hydrate chemotype, and the second belongs to the carvacrol/thymol type [56,57,58]. However, according to literature data gathered in this review paper, this is not always the case, as minor exceptions exist. For example, Chaves et al. (2020) [59] studied a sample of *O. majorana* originating from Brazil, which was found rich in pulegone (57.05%). Interestingly, no terpinen-4-ol or *cis*-sabinene hydrate or carvacrol were detected [59]. Furthermore, of the four studies that were found to analyze *O. majorana* from Morocco, one of them classified the studied sample as terpinen-4-ol chemotype (however without the second major in quantity compound being *cis*-sabinene hydrate) [60]; the other study identified the compound found in abundance as 4-terpinene [27], and in the rest of the studies, linalool (32.68%), sabinene hydrate (14.08%) and *trans*-sabinene hydrate (16.0%) were the most characteristic compounds [61,62,63]. However, remarkably, in the first two studies is the presence of terpinen-4-ol (22.30% and 13.07%, respectively).

Other studies that classified *O. majorana* to a different chemotype are those of Yang et al. (2009), Waller et al. (2016), Baj et al. (2018), Barazandeh et al. (2001) and Dantas et al. (2016) [64,65,66,67,68]. The first two studies [64,65] analyzed samples from India and Egypt, respectively, and found the major constituent being 1.8 cineole (50.96% and 20.9%, respectively). On the other hand, samples from Ukraine and Iran were rich in linalyl acetate (16.0% and 26.1%) [66,67]. Dantas et al. (2016) [68] studied a sample from Egypt. However, a different chemotype was observed, with **γ**-terpinene being the compound in abundance followed by α-terpinene.

*Origanum majorana* grown in Greece is classified into three chemotypes. Komaitis et al. (1992) [69] determined a terpinen-4-ol chemotype. This cyclic monoterpene constitutes 37.10% of the total content of essential oil, with *p*-cymene and α-terpineol being constituents that consist of 50% of the essential oil composition. Daferera et al. (2000) [57] also described an intermediate chemotype of thymol (14.0%) as the main compound. Carvacrol concentration reached 0.2%, while the other compounds found at higher concentrations were 3-carene (10.4%), 2-carene (7.8%), terpinen-4-ol (7.8%) and sabinene hydrate (6.0%). Finally, Giatropoulos et al. (2018) [19] identified a clear carvacrol chemotype, in which the concentration of carvacrol reached 74.8%.

In Table 1 is given summarized information about the collected literature data regarding the volatile profile of *O. majorana*. The most popular parts of the plant used are stems, flowers and leaves [33], from which essential oil is extracted mainly by steam distillation, a method adopted by the majority of researchers, as concluded from Table 1. Considerable variability is observed regarding the chemical composition of the plant, as well as the percentage yield of its essential oil. Terpinen-4-ol, *cis/trans*-sabinene hydrate, γ-terpinene, *cis*-β-terpineol, carvacrol and thymol are the compounds mentioned in abundance in the studied samples. Regarding the essential oil yield from the aerial parts of the plant, the % yield ranges from 0.4 to 1.85 mL/100 g of dry material, while when only leaves were used, the extent of the % yield ranges from 0.09 to 2.5 mL/100 g of dry material.

This chemical diversity of essential oil isolated from *O. majorana* samples is a product of different parameters such as the growth stage of the plant, climate variability, irrigated or arid crops, geographical area, soil salinity, storage conditions and method of distillation [70,71]. All these variables influence the production of secondary metabolites, thus affecting both the qualitative and quantitative composition of an essential oil. In particular, limited water availability is a factor that decreases crop yield and essential oil yield, or is even responsible for altering an essential oil composition.

A study conducted by Farsi et al. (2019) [72] examined the effect of partial irrigation on *O. majorana* crops. The authors evaluated three cases: sufficient irrigation of the crop, mild limited irrigation and moderate limited irrigation. Their results showed that inadequate water supply reduced both plant biomass and essential oil yield with respect to their control, fully irrigated crop; however, it did not affect the percentage of the compounds present in the essential oil [72]. Moreover, regarding the carvacrol chemotype, the high percentage of carvacrol can be attributed to the relative humidity at which the plant grows, wild or cultivated, or even the handling (dried or not) of the sample prior to analysis. The effect of humidity on carvacrol content was analyzed in the study of Bağci et al. (2017) [73]. The authors reported that the amount of this phenol-type compound on dried plant material was higher than that of fresh samples. In addition, the increase in carvacrol concentration was higher in the plants collected from the wild. A high concentration of carvacrol was also observed in cases of environmental aridity and increased the ambient temperature as well [74,75,76]. On the other hand, thymol abundance is correlated negatively with temperature, because an increase in thymols’ concentration is favored by a decrease in the ambient temperature. Carvacrol and thymol share a common biosynthetic pathway. The main precursor for the production of these two phenols is *γ*-terpinene, from which derives carvacrol via an oxidation reaction. Thymol is produced via hydroxylation of an intermediate, namely, *p*-cymene [77]. Therefore, it is usually the increased or decreased presence of *γ*-terpinene or/and *p*-cymene in marjoram essential oil classified as a carvacrol chemotype, because it is dependent on the increased or decreased percentage, respectively, of carvacrol and thymol.

As mentioned above, the most common chemotype is terpinen-4-ol accompanied in many cases by the presence of *cis*-sabinene hydrate. *Cis*-sabinene hydrate, together with *cis*-sabinene hydrate acetate, is considered the responsible compound for the sweet marjoram flavor [78]. Later, other authors reported that terpinen-4-ol also contributes to its characteristic aroma [79]. Nevertheless, *cis*-sabinene hydrate, rather than its acetate, is more frequently presented in marjoram essential oil. This may be because the acetate derivate is a less stable compound because of *cis*-sabinene hydrate resistance to temperature [80]. On the contrary, *cis*- and *trans*-sabinene hydrate are products of an enzymatic reaction catalyzed by sabinene hydrate synthase [81,82]. Furthermore, the authors of these studies stated that the production ratio of these compounds is 1 (*trans*-isomer):10 (*cis* isomer). However, this result was questioned by Novak et al. (2002) [80], who proposed that more enzymes must participate in this reaction, since according to their study, the ratio of 1:10 is not always stable.

**Table 1:** *Origanum majorana* essential oil from different geographic regions.

Plant Material	Extraction Method	Column Used for the GC Analysis	% Yield	Chemical Composition	Region	Reference
200 g of plant material (the part used is not identified)	Hydrodistillation (clevenger apparatus)	VB-5 30 × 0.25 mm, 0.25 μm	0.8 mL/100 g dry material	4-terpinene (28.96%), γ-terpinene (18.57%) and *α*-terpinene (12.72%), sabinene (8.02%)	Morocco	[27]
1000 g of the aerial parts	Hydrodistillation (according to European Pharmacopeia 5th edition guidelines)	DB-5 30 m × 0.25 mm, 0.33 μm	0.97 mL/100 g dry material	terpinen-4-ol (34.1%), *α*-terpinene (19.2%), terpineol (8.9%)	South West Morocco	[60]
10 g of plant material	Steam distillation (Likens–Nickerson apparatus)	CP-Sil 8 30 m, 0.32 mm	-	thymol (14.0%), 3-carene (10.4%), 2-carene (7.8%), terpinen-4-ol (7.8%), sabinene hydrate (6.0%)	Greece	[57]
100 g of aerial part (stems, leaves and flowers)	Hydrodistillation (clevenger apparatus)	HP-5MS 30 m × 0.25 mm, 0.25 μm	1.85 mL/100 g dry material	terpinen-4-ol (23.2%), *cis*-sabinene hydrate (17.5%), γ-terpinene (10.5%), p-cymene (9%), *α*-terpineol (5.6%)	Tunisia	[58]
100 g of leaves	Hydrodistillation (Quik-fit apparatus)	HP-5MS 30 m × 0.25 mm, 0.25 mm	0.09 mL/100 g dry material	terpinen-4-ol (555.1 μg/g dw), γ-terpinene (192.8 mg/g dw), *cis* sabinene hydrate (168.8 mg/g dw)	Tunisia	[35]
100 g of aerial parts) three developmental stages: vegetative, flowering and post-flowering)	Hydrodistillation (clevenger apparatus)	HP-5MS 30 m × 250 m, 0.25 μM	-	terpinen-4-ol (76.94–37.15), cyclohexanol 3,3,5 trimethyl (15.99–5.41), α-terpineol (11.34–0.94); β-cymene (10.56–1.88)	Tunisia	[47]
-	Steam distillation	Carbowax 20M 25 m × 0.3 mm	0.20 mL/100 g dry material	terpinen-4-ol (37.10%), p-cymene (12.05%), *α*-terpineol (7.15%)	Greece	[69]
Leaves	Hydrodistillation (clevenger apparatus)	DB-5MS 30 m × 0.25 mm × 0.25 μm	1.2 mL/100 g dry material	terpinen-4-ol (29.97%), γ-terpinene (15.40%), *trans*-sabinene hydrate (10.93%), *α*-terpinene (6.86%) and *α*-terpineol (6.54%)	Egypt	[14]
5 g	Hydrodistillation (clevenger apparatus)	Rtx-5MS 30 m × 0.25 mm × 0.25 μm	-	terpinen-4-ol (19.7%), *γ*-terpinene (18.4), *α*-terpinene (11.4%), *cis*-sabinene hydrate (8.6%), sabinene (7.8%)	Commercial sample Germany	[80]
Leaves	Hydrodistillation (clevenger apparatus)	DB5 30 m × 0.25 mm × 0.25 µm	-	carvacrol (57.86%), thymol (13.54%), *trans*-caryophyllene (11.52%), cymene (6.78%)	Iran	[49]
Aerial parts	Hydrodistillation (clevenger apparatus)	DB-5 30 m × 0.25 mm, 0.25 μm	-	terpinen-4-ol (31.15%), *cis*-sabinene hydrate (15.76%), p-cymene (6.83%), sabinene (6.91%), *trans*-sabinene hydrate (3.86%), *α*-terpineol (3.71%)	India	[36]
500 g of leaves	Hydrodistillation (clevenger apparatus)	HP-5MS 30 m × 0.25 mm, 0.25 μm	0.6 mL/100 g dry material	*cis*-sabinene hydrate (30.2%), terpinen-4-ol (28.8%), γ-terpinene (7.2%), *α*-terpineol (6.9%), *trans*-sabinene hydrate (4.4%), linalyl acetate (3.8%), *α*-terpinene (3.6%)	Venezuela	[79]
20 g of aerial part (two vegetative and two generative growth stages)	Hydrodistillation	HP-Innowax 30 m × 0.25 mm × 0.25 mm	0.04 to 0.09 mL/100 g dry material	terpinen-4-ol (29.13–32.57%), *cis*-sabinene hydrate (19.9–29.27%), *trans*-sabinene hydrate (3.5–11.61%), γ-terpinene (2.11–8.20%), bornyl acetate (1.52–2.94%), linalool (1.05–1.39%)	Tunisia	[37]
-	Hydrodistillation	Supelcowax 10, 60 m × 0.25 mm, 0.25 μm	0.8 mL/100 g dry material	terpinen-4-ol (30.3%), γ-terpinene (14%), linalool (12%), p-cymol (9.8%), *α*-pinene (5.9%), camphene (5.8%)	Hungary	[38]
Flowering plants	Hydrodistillation (clevenger apparatus)	Carbowax 20 M, 50 m × 0.32 mm i.d, 0.20 μm	1 mL/100 g dry material	terpinen-4-ol (38.4%), *cis*-sabinene hydrate (15.0%), p-cymene (7.0%), γ-terpinene (6.9%).	Reunion Island	[39]
-	Hydrodistillation (clevenger apparatus)	Equity-5 60 m × 0.32 mm, 0.25 μm	0.45–0.50 mL/100 g dry material	*cis*-sabinene hydrate (20.23–46.27%), terpinen-4-ol (9.32–23.43%), γ-terpinene (5.67–13.76%), *α*-terpinene (2.98–8.38%), sabinene (4.90–8.17%), *trans*-sabinene hydrate (5.01–7.34%), *α*-terpineol (3.41–4.17%)	India	[50]
Leaves	Hydrodistillation (clevenger apparatus)	DB-5 (5% phenylmethylpolysiloxane) capillary column, 60 m × 0.25 mm	1.6 mL/100 g dry material	terpinen-4-ol (30.0%), γ-terpinene (11.3%), *trans*-sabinene hydrate (10.8%)	Egypt	[26]
131 g leaves	Hydrodistillation (clevenger apparatus)	OPTIMAL-5 0.25 μm, 30 M, 0.25 mm	-	pulegone (57.05%), verbenone (16.92%), *trans*-menthone (8.57%)	Brasil	[60]
0.5 kg of aerial part	Hydrodistillation (clevenger apparatus)	HP-5 30 m × 0.25 mm, 0.25 μm	-	carvacrol (74.8%), thymol (2.7%)	Greece	[19]
-	-	DB-1MS 30 m × 0.25 mm, 0.25 µm	-	terpinen-4-ol (22.96%), linalool (15.32%), γ-terpinene (12.92%), p-cymene (6.37%)	Commercial sample, Korea	[24]
-	-	VF-5MS 30 m × 0.25 mm, 0.25 µm	-	terpinen-4-ol (33.8%), terpinolene (16.5%), linalool (14.7%), *α*-terpinene (6.8%)	Commercial sample	[28]
20 g dried leaves	Hydrodistillation (clevenger apparatus)	-	12.70 μL·g^−1^	terpinen-4-ol (23.83%) *cis*-β-terpineol (21.63%),	-	[20]
-	-	DB-5MS 30 m × 0.25 mm, 0.25 µm	-	1,8-cineole (50.96%), linalool (24.04%), limonene (6.38%)	India	[64]
Two samples from different regions were analyzed	Hydrodistillation	-	6.5–7.7 mL/100 g dry material	carvacrol (78.27–79.46%), p-cymene (4.31–4.68%), y-terpinene (3.72–4.84%)	Turkey	[56]
100 g of fresh plant material	Steam distillation	SE-54 50 m × 0.32 mm		linalool (32.68%), terpinen-4-ol (22.30%), p-cymene (8.07%)	Morocco	[61]
80 g of aerial parts	Hydrodistillation (clevenger apparatus)	HP-5MS 30 m × 0.25 mm, 0.25 µm	17.2 g/kg	terpinen-4-ol (20.9%), linalool (15.7%), linalyl acetate (13.9%), limonene (13.4%), *α*-terpineol (8.57%)	Pakistan	[40]
1 kg of dried aerial parts	Hydrodistillation	DB-5 30 m × 0.25 mm, 0.33 µm	0.4 mL/100 g dry material	terpinen-4-ol (29.6%), δ-2-carene (20.1%), camphene (13.4%), *α*-pinene (7.9%)	Italy	[12]
100 g dried aerial parts	Hydrodistillation (clevenger apparatus)	Cp WAX 52 CB 50 m × 0.32 mm, 1.2 µm	-	carvacrol (52.5%), linalool (45.4%),	Turkey	[51]
100 g of dried aerial part	▪ Microwave-assisted extraction (MWE) ▪ Hydrodistillation (HD) ▪ Steam distillation (SD)	TR-5 MS 30 m × 0.32 mm, 0.25 μm	(HD) 0.73 mL/100 g dry material (MWE) 0.80 mL/100 g dry material (SD) 0.66 mL/100 g dry material	terpinen-4-ol MWE: 22.28%, HD: 28.49%, SD: 26.72% *trans*-sabinene hydrate MWE: 13.05%, HD: 11.69%, SD: 3.04% γ-terpinene MWE: 13.20%, HD: 7.87%, SD: 13.72% *α*-terpinene MWE: 9.07%, HD 3.89%, SD: 9.46%	Egypt	[48]
300 g of plant material	Hydrodistillation (clevenger apparatus)	-	1.7 mL/100 g dry material	terpin-4-ol (27.32%), γ-terpinene (15.67%), α-terpinene (11.08%) *α*-terpineol (6.90%), sabinene (5.53%)	Tunisia	[52]
Aerial parts	Extraction with organic solvent	ZB-5MS (Phenomenex), 30 m × 0.25 mm, 0.25 µm		*trans*-sabinene hydrate (16.0%), sabinene (14.1%), *cis*-sabinene hydrate (11.8%), γ-terpinene (10.2%), *α*-terpinyl acetate (10.0%), *α*-terpinene (8.9%)	Yemen	[63]
-	-	DB-5 30 × 0.25 × 2.5 mm	-	terpinen-4-ol (20.55%), terpinene (13.13%), *trans*-terpineol (12.67%), 2-carene (7.67%), sabinene (6.96%)	-	[41]
-	-	ZB-5 MS 30 m, 0.25 mm, 0.25 μm	-	linalyl acetate (16.0%), linalool (14.7%), *α*-terpineol (13.8%), limonene (11.5%)	Commercial sample produced in Ukraine	[66]
-	Hydrodistillation (clevenger apparatus)	HP-5MS 30 m× 0.25 mm, 0.25 mm	-	terpinen-4-ol (32.69%), γ-terpinene (12.88%), *trans*-sabinene hydrate (8.47%), *α*-terpinene (7.98%), sabinene (6.21%)	-	[16]
200 g of aerial part	Hydrodistillation (Dean–Stark apparatus)	VB5 30 m × 0.25 mm 0.25 μm	1.06 mL/100 g dry material	Sabinene hydrate (14.08%), *α*-terpineol (13.95%), (-)-terpinen-4-ol (13.07%), (+)-sabinene (5.67%)	Morocco	[62]
-	-	HP-5 30 m× 0.32 mm× 0.25 mm	-	1,8-cineole (20.9%), terpinen-4-ol (20.4%), p-cymene (7.0%), sabinene (6.7%)	Commercial sample Egypt	[65]
Dried leaves	Hydrodistillation (clevenger apparatus)	DB-5 30 m × 0.25 mm 0.25 mm	1.20 mL/100 g dry material	terpinen-4-ol (30.41%), γ-terpinene (13.94%), *cis*-sabinene hydrate (9.64%), *α*-terpinene (7.70%)	Egypt	[53]
-	-	Restek 30 m × 0.32 mm, 0.50 μm	-	terpinen-4-ol (21.3%), *trans*-sabinene hydrate (15.5%), γ-terpinene (14.0%) and *α*-terpinene (8.9%)	Commercial product Albania	[54]
Aerial parts of plant material collected in different regions	Hydrodistillation (clevenger apparatus)	FSC 60 m × 0.25 mm, 0.25 µm	-	terpinen-4-ol (8–14%), linalyl acetate (7–10%), *trans*-sabinene hydrate (6–7%)	Turkey	[42]
100 g of air-dried aerial parts	Hydrodistillation (Dean–Stark apparatus)	HP-101 25 m × 0.32 mm	1.40 mL/100 g dry material	terpinen-4-ol (32.8%), y-terpinene (9.9%), *cis*-sabinene hydrate (8.6%)	Tunisia	[55]
Dried leaves	Hydrodistillation (clevenger apparatus)	TR-5MS 30 m × 0.25 mm, 0.25 μm	2.5 mL/100 g dry material	terpinen-4-ol (33.0%), caryophyllene oxide (11.9%), p-cymene (6.8%), *α*-terpineol (6.7%) spathulenol (6.0%)	Commercial sample China	[43]
200 g dried flowers 200 g dried leaves	Hydrodistillation (clevenger apparatus)	Supelcowax 10 30 m × 0.32 mm, 0.5 pm	12.8 mL/100 g dry material (flowers) 8% ml/100 g dry material (leaves)	Leaves: *cis*-sabinene hydrate (33.3%), terpinen-4-ol (21.6%), y-terpinene (8.3%), *α*-terpineol (7.3%), *trans*-sabinene hydrate (4.7% ) Flowers: *cis*-sabinene hydrate (24%), terpinen-4-ol (16.6%), *α*-terpineol (12.4%), y-terpinene (10.6%) Stems: terpinen-4-ol (19%), *α*-terpineol (14.25%), y-terpinene (11.1%), *cis*-sabinene hydrate (7.4%)	Cyprus	[70]
Flowers	Steam distillation	DB-1 60 m × 0.25 mm, 0.25 pm	0.3 mL/100 g dry material	linalyl acetate (26.1%), sabinene (12%), y-terpinene (8.8%), *cis*-sabinene hydrate (8.7%)	Iran	[67]
-	-	-	-	-	Egypt	[44]
-	-	DB-1 30 m × 0.25 mm, 0.25 μm	-	terpinen-4-ol (20.8%), γ-Terpinene (14.1%), cis-sabinene hydrate (10.8%) sabinene (9.3%), *α*-terpinene (9.2%)	Commercial sample UK	[45]
300 g of aerial parts	Hydrodistillation (clevenger apparatus)	-	1.72 mL/100 g dry material	terpinen-4-ol (26.7%), γ-terpinene (16.96%), p-menthenol (11.85%), *α*-terpinen (9.22%), *α*-terpineol (5.76%), p-cymene (5.27%)	Tunisia	[46]
Dried leaves	Hydrodistillation (clevenger apparatus)	Durabond-DB5 30 m × 0.25 mm × 0.25 μm	-	γ-terpinene (25.73%), α-terpinene (17.35%), terpinen-4-ol (17.24%), sabinene (10.8%), β-phellandrene	Egypt	[68]
200 g aerial part	(a) Microwave-assisted hydrodistillation (b) Hydrodistillation	HP-5 ms capillary 30 m × 0.25 mm, 0.25 μm	5 mL/100 g of dry material	(a) carvacrol (41.3%), linalool (12.2%), terpinen-4-ol (6.6%), linalyl acetate (6.8%), *γ*-terpinene (5.1%) (b) carvacrol (39.1%), linalool (7.2%), terpinen-4-ol (10.1%), linalyl acetate γ-terpinene (6.8%), (3.2%)	Greece	[71]
-	Hydrodistillation (clevenger apparatus)	-	0.2 mL/100 g of dry material	carvacrol (43.7%), thymol (18.3%), γ-terpinene (14.1%), *o*-cymene (8.1%), α-terpinene (2.0%)	Greece	[83]

In the studies mentioned in this review paper, the concentration of *cis*- and *trans*-sabinene hydrate ranges from 0.95% to 46.27%. This difference can be explained by taking into account the influence of abiotic components on the production of essential oils. As reported in the study of Novak et al. (2002) [80], increased temperature resulted in increased production of sabinene hydrate. In addition, apart from the effect of temperature, a longer period of sunlight had a positive influence on the production of *cis*- and *trans*-isomers, while the opposite was observed regarding the terpinene content [84]. Furthermore, row planting arrangement seems to be important. Single-row planting yielded essential oils richer in sabinene than binate rows. This effect was explained by the fact that single rows receive more sunlight [85]. On the other hand, the cyclic monoterpenes α- and *γ*-terpinene are frequently stated as components of the essential oil. Terpinen-4-ol, α-terpinene and *γ*-terpinene are typical products derived from a rearrangement reaction that follows the distillation process due to elevated temperature [86,87].

## 3. Insecticidal, Fumigant and Repellent Activity of *O. majorana* Essential Oil

Secondary metabolites are studied for their biological activity in an attempt to replace the use of synthetic compounds, since naturally derived products seem to relate with fewer side effects concerning human health. The field of study of secondary metabolites is multifarious. This review paper focuses on the studies dealing with the research being conducted in an attempt to replace chemical pesticides with essential oils.

The term pesticide includes different types of products, for example, insecticides, repellents, fungicides and many more. In fact, synthetic pesticides are currently used to protect crops and plants from insect pests. Although the effectiveness of these products is widely accepted, at the same time, concern is raised due to their toxicity because of pesticide residues in the crop. One crucial difference of an essential oil with a synthetic pesticide is the fact that due to its volatility, it is quickly degraded and thus does not remain on the surface of the final edible product. In addition, soil and water contamination are often completely avoidable [22].

*Origanum majorana* essential oil has been tested mainly against Lepidoptera such as *Spodoptera littoralis* Boisduval; *Ephestia kuehniella* Zeller; *Plodia interpunctella* Hübner; *Corcyra cephalonica* [20,26,29,30]; Coleoptera, including *Weevil Sitophilus* oryzae, *Tribolium castaneum* and *Sitophilus zeamais* Motschulsky [24,31,32]; Hemiptera such as *Apis fabae* L and *Myzus persicae* (Sulzer) [26,71]; and Tetranychidae such as *Tetranychus urticae* Koch [28]. The above-mentioned pests can infect either stored food or destroy crops. In both cases, the damage caused results in nutritional and economic losses. However, exposure of adults or larvae insects at different concentrations of essential oil resulted in population decline with high rates of mortality (Table 2). This effect is certainly attributed to the chemical composition of the essential oil and, of course, depends on the percentage and configuration of the compounds that are present. These compounds may have an additive effect. However, synergism or antagonism may also appear. A variation between the quantity of the oil used is observed, which is rather expected, since according to data given in Table 1, neither the composition of the essential oil nor the percentage of its constituents is the same. Therefore, to explain the biological activity, it is necessary to understand first the activity of every single compound of the essential oil, to explain their degradative behavior, to study their half-life and to explain their physicochemical properties. Unfortunately, such data are not yet clear or are very limited. For example, the European Food Safety Authority (EFSA), in their conclusion report on pesticide peer review [88], mentions the half-life of only three compounds, namely, α-terpinene, p-cymene and d-limonene.

In Table 3 is given information regarding the effect of pure compounds on insects’ survival. Data presented in this table refer to the concentration used against various types of insects, in order to examine their repellent, fumigant and insecticide activity. When the experiment refers to mortality, the concentration chosen to present here (when different doses were tested) was not that which had a 100% mortality. This derives from our thoughts that the lower the dose, the lower might be any other undesirable side effects (for example, toxicity to the plant or to beneficial insects for the crop). In addition, the rate of mortality for the adult insects given is that observed on the third day after treatment, since developmental delays or other developmental problems are better observed on this day. On the contrary, larvae insects’ mortality rates are given on the first day of treatment. As for the repellent activity, data are discussed at the shortest time of exposure and at the lowest concentration tested.

**Figure 1:**
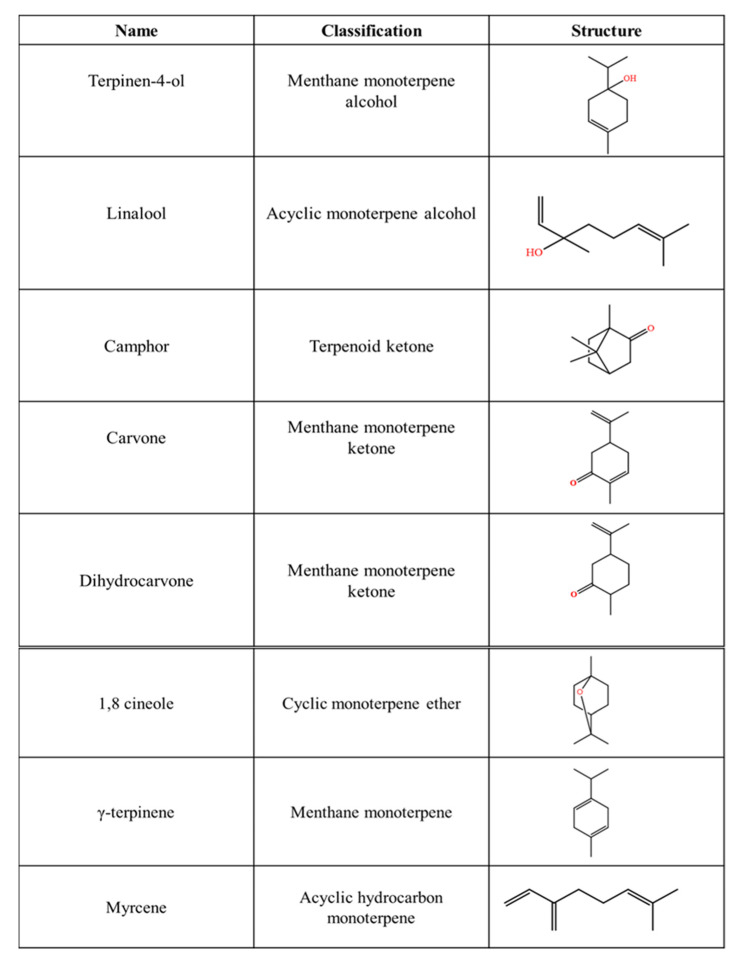
Terpenoids with insecticide, repellent and fumigant activity.

Comparing the data from Table 2 and Table 3, it is concluded that different doses are required in order for the essential oil or a pure compound to exert its pesticide activity. This is normally dependent not only on the targeted species or strain but also on the chemistry of the compound(s) used (as discussed below). Among the most prominent compounds in terms of their effect and the concentration used are terpinen-4-ol, linalool, camphor, carvone, dihydrocarvone, 1,8 cineole, γ-terpinene and myrcene. Structures and the terpene class to which these compounds belong are shown in Figure 1. For example, Abbassy et al. (2009) [26] studied the effect of γ-terpinene and terpinen-4-ol against *Spodoptera littoralis* Boisduval. γ-terpinene was more active than terpinen-4-ol, but both compounds acted synergistically with profenofos and methomyl, an organophosphate and a carbamate pesticide, respectively. The binary system used boosted the insecticidal activity of these two synthetic pesticides. Such synergistic activity was able to reduce the concentration of profenofos or methomyl, resulting in less harmful residues in food and the environment. *Tribolium castaneum* was found to be susceptible to myrcene, R(-)-carvone [23], terpinen-4-ol [90], carvacrol and thymol [83]. Volatiles have also been studied against the genus Sitophilus (*S. oryzae*, *S. granarius* (L.), *C. glomerata*, *P. xylostella* and *S. zeamais* Motschulsky [24,25,89,91,92]. The mortality rate of camphor, linalool, carvacrol, terpinen-4-ol, 1,8-cineole, carvone and dihydrocarvone, was high in the studied species and reached 100% for most of the compounds tested.

Taken together, the data presented here show that terpinen-4-ol was one of the compounds to almost always have a remarkable insecticidal or fumigant activity. Linalool, camphor and carvone are also worth mentioning. As can be seen in Figure 1, their activity, apart from the sensitivity of each species, is also dependent on the chemical structure of each compound. Kim et al. (2016) [24] reported that an aldehyde, ketone or alcohol group enhances the activity of a monoterpene. Consequently, compounds bearing the above-mentioned groups are more active with respect to monoterpenes hydrocarbons. Terpinen-4-ol and linalool belong both to tertiary alcohols. Both compounds were highly active against the insects tested. This is in accordance with the study of Seo et al. (2009) [93]; however, both these conclusions, are opposite to the observations of Choi et al. (2007) [94], who reported in their study that primary alcohols were the most active compounds regarding their nematicidal activity. Furthermore, it has been reported that aldehydes monoterpenes are more active than ketone monoterpenes. However, this is not always the rule, as according to data herein presented, camphor, carvone and dihydrocarvone are among the most active compounds [25,89]. Finally, the presence of an unsaturated α, β bond enhances the insecticidal activity of a compound, as in the case of linalool and carvone [95]. It remains to be clarified, however, why myrcene, an acyclic monoterpene bearing only methyl and methylene groups and γ-terpinene, a menthane monoterpene, presented better activity against camphor and linalool in inhibiting Tribonium castaneum [23] and *Spodoptera littoralis* Boisduval [26]. Maybe the membrane integrity and permeability of the insects are more prone to these compounds.

Data presented in this review paper are promising regarding the possible replacement of synthetic pesticides with those derived from natural sources. Nonetheless, some important issues should be addressed. For example, the use of essential oils as pesticides, apart from their decreased risk of adverse health effects, should at the same time outweigh the use of synthetic ones in effectiveness. In addition, their production must be economically more advantageous. Usually, the yield of *O. majorana* essential oil is low (from 0.4 to 1.85 mL/100 g, taking for example data extracted from this study). Consequently, a large amount of plant material is needed, and the most important is that the plant used should grow exactly under the same conditions and should be cropped in the same developmental stage. Thus, controlled environment agriculture should be used to grow plants. Furthermore, another issue to evaluate is the effect of the essential oil on crop development and on beneficial insects such as bees and butterflies. The studies mentioned here do not examine these parameters, which are of major importance. The toxic effect discussed focus on the capacity of the essential oil against insects’ lethality. In addition, few studies compare the activity of the essential oil with that of an appropriate control, namely, a synthetic pesticide, so as to extract more reliable conclusions [26]. Last but not least, duration of protection and solubility of the compounds in water or another environmentally friendly medium, should be evaluated [96]. The use of low concentrations of the isolated volatile compounds may simplify their dissolution in inorganic solvents.

## 4. Conclusions and Future Trends

Several research teams have examined the volatile profile of *Origanum majorana*. Steam distillation is the technique most used to collect the essential oil; however, a considerable variation between the studies was revealed, which is attributed to the plant itself (i.e., developmental stage) or the different geographic areas, which means, at the same time, different climatic and soil conditions. Despite this variability, and regardless of the constituent present in abundance, the essential oil from *Origanum majorana* possesses considerable insecticide activity. Indeed, such biological activity is of paramount importance. Given the exposure of humans and the environment to pesticide residues, a great effort is made to replace synthetic pesticides with natural and consequently less harmful ones. *Origanum majorana* is one promising example of such possible use.

## Figures and Tables

**Table 2:** Activity of *Origanum majorana* essential oil against agricultural insects.

Species Examined	Family/Order	Dose Used	Effect	Reference
*Corcyra cephalonica*	Pyralidae/Lepidoptera	11.31 μL/L air (adult) 49.83 μL/L air (larvae)	Fumigant toxicity	[20]
*Spodoptera littoralis* Boisduval	Noctuidae/Lepidoptera	2.48 μg 3.14 g/L 1.86 g/L 2.27 g/L	Insecticidal activity	[26]
*Aphis fabae* L.	Aphididae/Hemiptera			
*Tetranychus urticae* Koch	Tetranychidae/Tetranychidae	15 μg/cm^2^	Fumigant activity	[28]
*Ephestia kuehniella* Zeller	Pyralidae/Lepidoptera	200 μg/L air 200 μg/L air	Fumigant activity	[29]
*Plodia interpunctella* (Hübner)	Pyralidae/Lepidoptera			
*Spodoptera littoralis*	Noctuidae/Lepidoptera	19.6 mL/m^3^ LD50	Insecticidal activity	[30]
*Tribolium castaneum*	Tenebrionidae/Coleoptera	100 ppm	Repellent activity	[31]
*Sitophilus zeamais Motschulsky*	Curculionidae/Coleoptera	0.18 mg/cm^2^	Insecticidal activity	[32]
*Myzus persicae* (Sulzer)	Aphididae/Hemiptera	500 μL/L	Longevity and fecundity	[71]
*Tribolium. castaneum* (Herbst) *Trogoderma granarium* (Everts)	Tenebrionidae/Coleoptera Dermestidae/Coleoptera	1000 ppm	Insecticidal activity	[83]

**Table 3:** Activity of pure compounds against agricultural insects.

Species Examined Method of Exposure	Compound Name	Dose Requested for the Activity and % Effect after the Application (in Parenthesis)		Reference
*Spodoptera littoralis* Boisduval Topical application/Residual film	terpinen-4-ol	16.20 μg/larva	32.94 g/L	[26]
	*γ*-terpinene	11.86 μg/larva	23.94 g/L	
*RapidAphis fabae* L. Rapid dipping/Residual film	terpinen-4-ol	14.86 g/L	20.77 g/L	
	*γ*-terpinene	12.24 g/L	18.03 g/L	
*Sitophilus oryzae* filter paper disc	camphene	6.5 mg/L (10%)		[24]
	*α*-terpinene	25 mg/L (52%)		
	sabinene hydrate	6.5 mg/L (26%)		
	terpinolene	25 mg/L (98%)		
	linalool	6.5 mg/L (74%)		
	camphor	6.5 mg/L (22%)		
	*α*-terpineol	1.5 mg/L (18%)		
	terpinen-4-ol	3 mg/L (88%)		
*Rhyzopertha dominica* filter paper disc	1,8-cineole	0.1 μL/720 mL (97.5%)		[89]
	linalyl acetate	0.1 μL/720 mL (90%)		
	carvacrol	0.1 μL/720 mL (82.5%)		
	camphor	0.1 μL/720 mL (100%)		
	linalool	0.1 μL/720 mL (100%)		
	bornyl acetate	0.1 μL/720 mL (92.5%)		
	borneol	0.1 μL/720 mL (92.5%)		
	thymol	0.1 μL/720 mL (77.5%)		
	linalyl acetate	0.1 μL/720 mL (0.00%)		
*Tribolium castaneum* filter paper disc	1,8-cineole	0.1 μL/720 mL (0.00%)		
	carvacrol	0.1 μL/720 mL (5.0%)		
	camphor	0.1 μL/720 mL (0.00%)		
	linalool	0.1 μL/720 mL (0.00%)		
	bornyl acetate	0.1 μL/720 mL (0.00%)		
	borneol	0.1 μL/720 mL (0.00%)		
	thymol	0.1 μL/720 mL (0.00%)		
*Sitophilus oryzae* filter paper disc	linalyl acetate	0.1 μL/720 mL (100%)		
	1,8-cineole	0.1 μL/720 mL (100%)		
	carvacrol	0.1 μL/720 mL (85%)		
	camphor	0.1 μL/720 mL (90%)		
	linalool	0.1 μL/720 mL (90%)		
	bornyl acetate	0.1 μL/720 mL (97.5%)		
	borneol	0.1 μL/720 mL (100%)		
	thymol	0.1 μL/720 mL (100%)		
*Tribolium castaneum* surface-film bioassay (contact toxicity)	terpinen-4-ol	0.21 mg/cm^2^		[90]
	*α*-terpinene	>0.50 mg/cm^2^		
	p-cymene	>0.50 mg/cm^2^		
*Tribolium castaneum* surface-film bioassay (fumigant toxicity)	terpinen-4-ol	20.47 mg/cm^2^		
	*α*-terpinene	23.70 mg/cm^2^		
	p-cymene	27.01 mg/cm^2^		
*Tribolium castaneum* area preference method (repellent activity)	terpinen-4-ol	0.001 mg/cm^2^ (23.3% RI ^1^, 2 h)		
	*α*-terpinene	0.001 mg/cm^2^ (80.0%% RI ^1^, 2 h)		
	*p*-cymene	0.001 mg/cm^2^ (66.70%% RI ^1^, 2 h)		
*Plutella xylostella* vapor-phase mortality bioassay (fumigant toxicity)		JJ-PX *P. xylostella* larvae	KS-PX *P. xylostella* larvae	[91]
	linalool	0.021 mg cm^−3^	0.016 mg cm^−3^	
	linalool oxide	0.024 mg cm^−3^	0.016 mg cm^−3^	
	terpinen-4-ol	0.020 mg cm^−3^	0.018 mg cm^−3^	
	(1S)-(−)-camphor	0.022 mg cm^−3^	0.019 mg cm^−3^	
	(1R)-(+)-camphor	0.029 mg cm^−3^	0.024 mg cm^−3^	
	1.8-cineole	0.029 mg cm^−3^	0.037 mg cm^−3^	
	*p*-cymene	0.037 mg cm^−3^	0.038 mg cm^−3^	
	(1R)-(+)-α-pinene	0.047 mg cm^−3^	0.045 mg cm^−3^	
	(1S)-(−)-α-pinene	0.040 mg cm^−3^	0.052 mg cm^−3^	
	(1R)-(+)-β-Pinene	0.058 mg cm^−3^	0.046 mg cm^−3^	
	(1S)-(−)-β-Pinene	0.063 mg cm^−3^	0.057 mg cm^−3^	
	camphene	0.060 mg cm^−3^	0.074 mg cm^−3^	
	α-Terpineol	0.069 mg cm^−3^	0.076 mg cm^−3^	
	(R)-(−)-α-Phelladrene	0.109 mg cm^−3^	0.087 mg cm^−3^	
	(1S)-(−)-Borneol	0.140 mg cm^−3^	0.121 mg cm^−3^	
	(1R)-(+)-Camphor	0.029 mg cm^−3^	0.024 mg cm^−3^	
*Cotesia glomerata* vapor-phase mortality bioassay (fumigant toxicity)	(1S)-(−)-Camphor	0.0016 mg cm^−3^		
	terpinen-4-ol	0.0018 mg cm^−3^		
	1.8-cineole	0.0039 mg cm^−3^		
	bornyl acetate	0.0064 mg cm^−3^		
	linalool	0.0075 mg cm^−3^		
	α-terpineol	0.0078 mg cm^−3^		
	(1S)-(−)-β-pinene	0.0083 mg cm^−3^		
	α-Terpinyl acetate	0.0084 mg cm^−3^		
	(1R)-(+)-β-Pinene	0.0089 mg cm^−3^		
	(1R)-(+)-α-Pinene	0.018 mg cm^−3^		
	nerol	0.0093 mg cm^−3^		
	neryl acetate	0.012 mg cm^−3^		
	linalyl acetate	0.014 mg cm^−3^		
	camphene	0.015 mg cm^−3^		
	(1S)-(−)-α-Pinene	0.015 mg cm^−3^		
	geranyl acetate	0.016 mg cm^−3^		
	limonene	0.016 mg cm^−3^		
	γ-Terpinene	0.017 mg cm^−3^		
	β-Caryophyllene	0.018 mg cm^−3^		
	myrcene	0.019 mg cm^−3^		
	p-cymene	0.021 mg cm^−3^		
	(R)-(−)-α-phelladrene	0.025 mg cm^−3^		
	limonene	0.027 mg cm^−3^		
	α-Terpinene	0.026 mg cm^−3^		
	geraniol	0.032 mg cm^−3^		
*Cotesia glomerata* leaf-dip bioassay (residual toxicity)		JJ-PX *P. xylostella* larvae	KS-PX *P. xylostella* larvae	
	terpinen-4-ol	0.0538 mg cm^−2^	0.0405 mg cm^−2^	
	linalool	0.0582 mg cm^−2^	0.0489 mg cm^−2^	
	linalool oxide	0.0654 mg cm^−2^	0.0521 mg cm^−2^	
	(1S)-(−)-camphor	0.0816 mg cm^−2^	0.0737 mg cm^−2^	
	*p*-cymene	0.1586 mg cm^−2^	0.1388 mg cm^−2^	
	1,8-cineole	0.1726 mg cm^−2^	0.1552 mg cm^−2^	
	(1R)-(+)-α-pinene	0.4996 mg cm^−2^	0.4486 mg cm^−2^	
*Tribolium castaneum* (Herbst) (area preference method)	myrcene	2 × 10^−5^ μL/cm^2^ (8%)		[23]
	carvacrol	2 × 10^−5^ μL/cm^2^ (8%)		
	geraniol	2 × 10^−5^ μL/cm^2^ (6%)		
	geranyl acetate	2 × 10^−5^ μL/cm^2^ (5%)		
	nerol	2 × 10^−5^ μL/cm^2^ (8%)		
	p-cymene	2 × 10^−5^ μL/cm^2^ (−6%)		
	R(-) carvone	2 × 10^−5^ μL/cm^2^ (21%)		
	S(+) carvone	2 × 10^−5^ μL/cm^2^ (−10%)		
*Sitophilus granarius* (L.) (petri dishes)	camphene	10 μg/petri dish (22.2%)		[25]
	3-carene	8.7 μg/petri dish (88.9%)		
	limonene	8.4 μg/petri dish (91.9%)		
	myrcene	7.9 μg/petri dish (79.8%)		
	*γ*-terpinene	8.5 μg/petri dish (80.8%)		
	borneol	10 μg/petri dish (54.5%)		
	linalool	8.6 μg/petri dish (100%)		
	thymol	10 μg/petri dish (73.7%)		
	carvacrol	9.8 μg/petri dish (100%)		
	nerol	8.8 μg/petri dish (100%)		
	terpinen-4-ol	10 μg/petri dish (100%)		
	*α*-terpineol	10 μg/petri dish (62.6%)		
	1.8-cineole	9.2 μg/petri dish (100%)		
	camphor	10 μg/petri dish (49.5%)		
	carvone	9.6 μg/petri dish (100%)		
	dihydrocarvone	9.3 μg/petri dish (100%)		
*Sitophilus zeamais* Motschulsky (petri dishes)	camphene	10 μg (4.04%)		[92]
	3-carene	10 μg (47.47%)		
	limonene	10 μg (6.06%)		
	myrcene	10 μg (4.04%)		
	*α*-pinene	10 μg (4.04%)		
	*β*-pinene	10 μg (3.03%)		
	linalool	10 μg (76.77%)		
	nerol	10 μg (3.03%)		
	terpinen-4-ol	10 μg (96.97%)		
	*α*-terpineol	10 μg (45.45%)		
	1.8-cineole	10 μg (89.90%)		
	camphor	10 μg (75.76%)		
	carvone	10 μg (100%)		
	dihydrocarvone	10 μg (100%)		

^1^ RI: repellent activity.
